# Racial Disparities in the Clinical Prognosis of Gastrointestinal Cancer Patients with COVID-19: a Retrospective Study in UC CORDS

**DOI:** 10.1007/s40615-023-01512-w

**Published:** 2023-01-13

**Authors:** Bingya Ma, Yunxia Lu

**Affiliations:** Department of Population Health and Disease Prevention, Program in Public Health, Susan and Henry Samueli College of Health Sciences, University of California, Irvine, CA USA

**Keywords:** Racial groups, Gastrointestinal cancer, COVID-19, SARS-COV-2, Vaccination

## Abstract

**Background:**

Cancer patients are highly vulnerable to the severe acute respiratory syndrome coronavirus 2 (SARS-CoV-2) infection. Few studies have examined racial disparities of clinical prognosis among gastrointestinal (GI) cancer patients with COVID-19, especially after the approval of COVID-19 vaccines.

**Methods:**

We conducted a retrospective study based on the University of California COVID Research Data Set (UC CORDS). Patients aged ≥ 18 with GI cancer as well as SARS-CoV-2 infection between March 10, 2020, and May 8, 2022, were included. We examined racial disparities using multivariable logistic regression.

**Results:**

Among the 1054 GI cancer cases included, 117 (11.1%) patients were Asian and Pacific Islander, 51 (4.8%) were Black patients, 377 (35.8%) were Hispanic patients, 403 (38.2%) were White patients, and 106 (10.1%) belonged to other or unknown races. Fully adjusted logistic models revealed a significantly increased risk of COVID-19-related hospitalization or emergency room visits among the Black (OR = 2.26, 95% CI = 1.08–4.70), the Hispanic (OR = 2.24, 95% CI = 1.48–3.39), and the patients of other or unknown races (OR = 1.80, 95% CI = 1.00–3.26) compared with the White patients. No significant racial disparities in 30-day all-cause mortality and mechanical ventilation rate were found. Vaccination, age, cancer type, recent cancer diagnoses in UC CORDS, metastatic cancer or secondary malignant neoplasm, and Charlson comorbidity index score were associated with the prognosis of GI cancer patients with COVID-19.

**Conclusions:**

GI cancer patients belonging to racial minorities experience worse COVID-19 outcomes. Vaccination status is a crucial factor associated with GI cancer patients’ prognosis among different race/ethnicity groups. Targeted communication in the context of cancer is needed to encourage vaccination uptake in this vulnerable population.

**Supplementary Information:**

The online version contains supplementary material available at 10.1007/s40615-023-01512-w.

## Introduction

Coronavirus disease 2019 (COVID-19) is an infectious disease caused by severe acute respiratory syndrome coronavirus 2 (SARS-CoV-2) and has quickly turned into a global pandemic since March 2020 [1]. Cancer patients are highly vulnerable to SARS-CoV-2 infection with a high hospitalization rate and mortality, likely due to a systemic immunosuppressive condition caused directly by tumor growth or indirectly by the side effects of anticancer therapy [2]. A few epidemiological studies have investigated the racial disparities in clinical prognosis among cancer patients with COVID-19. A significantly increased risk of hospitalization and mortality for patients with cancer in general [3–6] or specific cancer (e.g., breast [7], gynecological [8], hematological [9], and prostate cancer [10]) was reported among racial minorities including African Americans (AA) or Blacks, American Indians and Alaska Natives (AI/AN), Asians and Pacific Islanders (API), and Hispanics.

Different from other cancers, the incidence of GI cancer demonstrates distinct racial disparities associated with the etiology of cancer. Infection-related GI cancer (e.g., esophageal squamous cell carcinoma, gastric cancer, and hepatitis-related liver cancer) is predominant among API, Hispanic, and AI/AN people who make up around 60% of the total population in California [11]; however, obesity-related cancer (e.g., colorectal cancer, small intestinal cancer, pancreatic cancer, and obesity-related liver cancer) is more prevalent among the Black, the AI/AN and the Non-Hispanic White [12]. Although overall cancer incidence has been decreasing in the U.S. population, several GI cancers (e.g., liver cancer and young-onset colorectal cancer) continue to increase, especially among racial minorities [13, 14]. Risk factors of GI cancer such as infection (e.g., *Hepatitis B* virus, *Heliobacter pylori*), lifestyle (e.g., obesity), and preventive parameters (e.g., participation in screening for colorectal cancer) are distributed differently by race/ethnicity groups that may contribute to racial disparities of cancer incidence and prognosis.

The disparate impact of the COVID-19 pandemic on racial minorities has been widely reported over the past two years. However, studies on GI cancer are sparse, especially after the approval of COVID-19 vaccines in December 2020, although GI cancer is one of the major cancer types in California [15, 16]. An investigation of racial disparities in the prognosis of GI cancer patients with COVID-19 is warranted.

## Methods

### Study Population

This is a retrospective study based on the University of California COVID Research Data Set (UC CORDS). UC CORDS is a limited dataset containing structured electronic health records (EHRs) of individuals tested for COVID-19 across the five UC medical campuses (Davis, San Francisco, Los Angeles, Irvine, and San Diego), regardless of the test results [17]. EHRs are connected via the Observational Medical Outcomes Partnership Common Data Model (OMOP CDM), standardizing the underlying clinical coding systems, and providing a uniform healthcare data format [18]. Patients aged ≥ 18 with GI cancer (malignancies of the esophagus, the stomach, the small and large intestine, the anus, the pancreas, the liver, and the biliary system) and confirmed SARS-CoV-2 infection between March 10, 2020, and May 8, 2022, were included in this study. SARS-CoV-2 infection was based on positive results of real-time reverse transcriptase polymerase chain reaction (RT-PCR) assays of specimens. Patients might have multiple tests or infections, but only the first episodes of their infections were included in this study. The infection date was defined as the measurement date of the first positive result. The third edition of the International Classification of Diseases for Oncology (ICD-O-3) was used to identify GI cancer as C150-C269. International Classification of Diseases 10th Revision (ICD-10) and 9th Revision were also utilized as C15-C26 and 150–157, 159, respectively, to supplement GI cancer identification. Standard concept IDs of GI cancer in OMOP CDM were retrieved according to the above ICD codes and applied to identify the study cohort. The use of the dataset was jointly reviewed by the Institutional Review Boards of all University of California Health campuses and was determined to be a non-human subject’s research.

### Definition of Race/Ethnicity and Outcomes

The observed patients were categorized into five groups based on racial identity according to self-reported data in EHR with the combination of OMOP CDM standard concept IDs: the API (race: 8515, 8557 and ethnicity: 38,003,564), the Black (race: 8516 and ethnicity: 38,003,564), the Hispanic (ethnicity: 38,003,563), the White (race: 8527 and ethnicity: 38,003,564), and other or unknown races (other combinations of standard concept IDs). The three study outcomes that were identified within 30 days of SARS-CoV-2 infection were as follows: (1) all-cause mortality, (2) mechanical ventilation, and (3) COVID-19-related hospitalization or emergency room (ER) visits. COVID-19-related hospitalization or ER visits were defined as hospitalization or ER visits with COVID-19 as one of the EHR encounter diagnoses. We also explored whether the racial disparities in COVID-19 outcomes were associated with health-related factors, e.g., vaccination status, body mass index (BMI), or cancer-related factors, e.g., cancer type and stage.

### Covariates

Covariates included age, sex, BMI, Charlson comorbidity index (CCI) score, cancer type, cancer stage, recent cancer diagnoses in UC CORDS, COVID-19 vaccination status, and dominant SARS-COV-2 variant at COVID-19 diagnosis. As the exact date of birth was not available in UC CORDS due to de-identification, we calculated age by subtracting the year of birth from the year of SARS-COV-2 infection. BMI was defined as the most recent weight before the SARS-COV-2 infection in kilograms divided by height in meters squared. We further categorized age and BMI into several groups to describe patients’ baseline characteristics. They were used as continuous variables in regression analysis because of the better goodness of fit in the regression model. We calculated CCI scores according to the algorithm developed by Glasheen et al. [19]. Since all study subjects were GI cancer patients, we removed the GI cancer diagnosis from the calculation of the CCI score. We created a proxy variable of cancer stage using metastatic cancer or secondary malignant neoplasm data because the cancer stage was unavailable in the UC CORDs. GI cancer was classified into four categories: (1) GI tract cancer (malignancies of the esophagus, the stomach, the small and large intestine, and the anus), (2) hepatobiliary or pancreatic cancer, (3) other or ill-defined GI cancer, and (4) multiple GI cancers (combinations of more than two of other three categories). Recent cancer diagnoses in UC CORDS were determined by the time when cancer diagnoses were first recorded or reported by the patients in the UC Health system and was categorized as yes (for those diagnosed within one year of SARS-COV-2 diagnosis) and no (for those diagnosed more than a year before SARS-COV-2 diagnosis) in the analysis. Dominant SARS-COV-2 variants at COVID-19 diagnosis was also categorized based on the time of SARS-COV-2 infection: original types before Delta variant (before June 12, 2021), Delta (between June 12, 2021, and December 20, 2021), and Omicron (after December 20, 2021) [20]. We categorized vaccination status into three groups: unvaccinated, partially vaccinated (received only one dose of mRNA vaccines), and fully vaccinated (received at least two doses of mRNA vaccines or one dose of Ad26 vector-based vaccines).

### Statistical Analysis

Descriptive statistics were used to summarize the baseline demographics, health-related, and cancer-related information according to the clinical outcomes of the patients. Numerical variables were presented as medians and interquartile ranges (IQRs), and categorical variables were reported as frequencies and proportions. Racial disparities in study subjects were further analyzed by minimally, moderately, and fully adjusted multivariable logistic regression models. The minimally adjusted model (adjusted model 1) was controlled by age and sex. Any covariable whose univariate test had a *p* value < 0.1 was a candidate for backward and stepwise selection. Variables selected by backward and stepwise methods, along with age and sex, were included in the moderately adjusted model (adjusted model 2). The fully adjusted model contained the nine covariates mentioned in the previous section (see the “Covariates” section, adjusted model 3). We calculated odds ratios (ORs) and 95% confidence intervals (CIs) of specific race/ethnicity groups compared to the White patients using logistic regression models. Due to the limited sample size and clinical events in certain racial groups, we combined data of the Black people with other or unknown races when all-cause mortality and mechanical ventilation were the outcomes.

Some patients had no height or weight values recorded, most likely due to infrequent medical visits before the COVID-19 diagnosis. We handled the missing values of height and weight data using imputation analyses. Sensitivity analyses were further conducted to check the robustness of the primary findings by excluding patients with missing height and weight information. To avoid bias caused by weight change among cancer patients, we also conducted sensitivity analyses by excluding patients with weight measured more than 3 months before COVID-19 diagnosis. Furthermore, we did a subgroup analysis to check whether the primary findings were consistent before and after the Omicron variant of SARS-COV-2 became the dominant strain of the virus circulating in California. We did all analyses using Statistical Analysis System (SAS 9.4, SAS Institute, North Carolina). Statistical tests were 2-sided, and a *p* value < 0.05 was considered statistically significant.

## Results

### Patient Characteristics

Among 90,907 patients with a SARS-COV-2 test in the UC CORDS dataset between March 10, 2020, and May 8, 2022, 1228 GI cancer patients tested positive for SARS-COV-2. Of these, we excluded 165 patients diagnosed with GI cancer after SARS-COV-2 infection and nine patients under the age of 18. Ultimately, 1054 patients remained in the analysis. Among them, 117 (11.1%) were the API, 51 (4.8%) were Black, 377 (35.8%) were Hispanic, 403 (38.2%) were White, and 106 (10.1%) of other or unknown races. The median age of the study population was 64 years (IQR 55–73 years), and most patients were men (*N* = 611, 58.0%).

Characteristics of the patients by race, as recorded within 30 days of COVID-19 infection, are shown in Table 1. Hispanic patients were younger [median age 61 years (IQR 52–69 years)] compared with patients of other races. Black and Hispanic patients had a higher rate of obesity (37.3% and 28.6%), metastasis or secondary malignant neoplasm (74.5% and 67.1%), and more comorbidities than other racial groups. Regarding the cancer type, the proportion of GI tract cancer was higher among Black (70.6%) and White (63.0%) patients. In comparison, hepatobiliary and pancreatic cancer was more prevalent among API (42.7%) and Hispanics (49.3%). As for COVID-19-related characteristics, 88.0% of the GI patients were infected with SARS-COV-2 when Delta and Omicron were the dominant variants. Furthermore, the rate of fully vaccinated was 21.8% in all of the patients before the first positive SARS-CoV-2 test, while the rate of fully vaccinated in the Hispanic patients was only 14.6%.

**Table 1:** Baseline characteristics of GI cancer patients with SARS-CoV-2 infection in 30 days by race

Variable	Asian or Pacific Islander (*N* = 117 [11.1%])	Black (*N* = 51 [4.8%])	Hispanic (*N* = 377 [35.8%])	White (*N* = 403 [38.2%])	Other or unknown races (*N* = 106 [10.1%])	Total (*N* = 1054 [100.0%])
Age, median (IQR), years	66.0 (58.0–77.0)	65.0 (58.0–73.0)	61.0 (52.0–69.0)	67.0 (57.0–75.0)	64.5 (56.0–71.0)	64.0 (55.0–73.0)
Age group (years)						
18–64	52 (44.4%)	25 (49.0%)	238 (63.1%)	174 (43.2%)	53 (50.0%)	542 (51.4%)
≥ 65	65 (55.6%)	26 (51.0%)	139 (36.9%)	229 (56.8%)	53 (50.0%)	512 (48.6%)
Sex						
Female	54 (46.2%)	23 (45.1%)	171 (45.4%)	147 (36.5%)	48 (45.3%)	443 (42.0%)
Male	63 (53.8%)	28 (54.9%)	206 (54.6%)	256 (63.5%)	58 (54.7%)	611 (58.0%)
BMI median (IQR), kg/m^2^	23.8 (20.8–26.8)	26.8 (23.9–35.1)	26.8 (23.6–30.9)	26.1 (22.9–29.7)	25.4 (22.1–28.6)	26.1 (22.9–29.9)
BMI, kg/m^2^						
< 25	64 (54.7%)	16 (31.4%)	126 (33.4%)	152 (37.7%)	45 (42.5%)	403 (38.2%)
≥ 25 and < 30	35 (29.9%)	14 (27.5%)	131 (34.7%)	146 (36.2%)	36 (34.0%)	362 (34.3%)
≥ 30	12 (10.3%)	19 (37.3%)	108 (28.6%)	86 (21.3%)	17 (16.0%)	242 (23.0%)
Missing or unknown	6 (5.1%)	2 (3.9%)	12 (3.2%)	19 (4.7%)	8 (7.5%)	47 (4.5%)
CCI score						
0–2	48 (41.0%)	14 (27.5%)	134 (35.5%)	189 (46.9%)	53 (50.0%)	438 (41.6%)
≥ 3	69 (59.0%)	37 (72.5%)	243 (64.5%)	214 (53.1%)	53 (50.0%)	616 (58.4%)
Cancer type						
GI tract cancer	64 (54.7%)	36 (70.6%)	172 (45.6%)	254 (63.0%)	61 (57.5%)	587 (55.7%)
Hepatobiliary or pancreatic cancer	50 (42.7%)	14 (27.5%)	186 (49.3%)	134 (33.3%)	39 (36.8%)	423 (40.1%)
Multiple GI cancers	3 (2.6%)	1 (2.0%)	16 (4.2%)	11 (2.7%)	6 (5.7%)	37 (3.5%)
Other or ill-defined GI cancer	0 (0.0%)	0 (0.0%)	3 (0.8%)	4 (1.0%)	0 (0.0%)	7 (0.7%)
Metastasis or secondary malignant neoplasm						
Yes	47 (40.2%)	13 (25.5%)	124 (32.9%)	152 (37.7%)	38 (35.8%)	374 (35.5%)
No	70 (59.8%)	38 (74.5%)	253 (67.1%)	251 (62.3%)	68 (64.2%)	680 (64.5%)
Recent cancer diagnoses in UC CORDS						
Yes	54 (46.2%)	19 (37.3%)	160 (42.4%)	171 (42.4%)	43 (40.6%)	447 (42.4%)
No	63 (53.8%)	32 (62.7%)	217 (57.6%)	232 (57.6%)	63 (59.4%)	607 (57.6%)
Dominant SARS-CoV-2 variant at COVID-19 diagnosis						
Original types before Delta	10 (8.5%)	2 (3.9%)	43 (11.4%)	57 (14.1%)	14 (13.2%)	126 (12.0%)
Delta	61 (52.1%)	21 (41.2%)	121 (32.1%)	192 (47.6%)	46 (43.4%)	441 (41.8%)
Omicron	46 (39.3%)	28 (54.9%)	213 (56.5%)	154 (38.2%)	46 (43.4%)	487 (46.2%)
Vaccination status						
Unvaccinated	82 (70.1%)	37 (72.5%)	310 (82.2%)	269 (66.7%)	80 (75.5%)	778 (73.8%)
Partially vaccinated	5 (4.3%)	2 (3.9%)	12 (3.2%)	22 (5.5%)	5 (4.7%)	46 (4.4%)
Fully vaccinated	30 (25.6%)	12 (23.5%)	55 (14.6%)	112 (27.8%)	21 (19.8%)	230 (21.8%)

*IQR* interquartile ranges, *BMI* body mass index, *CCI* Charlson comorbidity index

### Racial Disparities in Clinical Prognosis

Among 1054 included patients, 74 (7.0%) patients died, 45 (4.3%) required mechanical ventilation, and 203 (19.3%) were hospitalized or admitted to ER with COVID-19 as one of the EHR encounter diagnoses within 30 days of COVID-19 infection. Tables 2 and 3 display the odds ratios and 95% CIs of specific races relative to that of the White patients for different outcomes. Compared to the non-Hispanic White patients, no statistically significant racial differences in all-cause mortality were detected in almost all unadjusted and adjusted models (Table 2). Hispanic patients had a 2.04 times risk of mechanical ventilation in the unadjusted model (OR = 2.04, 95% CI = 1.02–4.07) and the model adjusted for age and sex (OR = 2.55, 95% CI = 1.26–5.18) (Table 2). However, we observed no statistically significant racial differences for mechanical ventilation when the model was adjusted for statistically selected variables, including CCI score and vaccination status (OR = 1.91, 95% CI = 0.93–3.93) and the fully adjusted model (OR = 2.02, 95% CI = 0.95–4.30) (Table 2). In the unadjusted models, we observed an enhanced risk of 30-day COVID-19-related hospitalization or ER visits across all racial minority groups compared to the non-Hispanic White (Table 3). The fully adjusted logistic models also revealed a significantly increased risk of COVID-19-related hospitalization or ER visits among the Black patients (OR = 2.26, 95% CI = 1.08–4.70), the Hispanic patients (OR = 2.24, 95% CI = 1.48–3.39), and the patients of other or unknown races (OR = 1.80, 95% CI = 1.00–3.26) when compared with the White patients (Table 3). Similar results were shown in sensitivity analyses, excluding patients with missing height and weight values or weight measured more than 3 months before COVID-19 diagnosis (Supplementary Table 1).

**Table 2:** Odds ratios and 95% CIs of specific races relative to the White by the outcomes of all-cause mortality or mechanical ventilation in GI cancer patients with SARS-CoV-2 infection in 30 days

Outcome	White (*N* = 403)	Asian or Pacific Islander (*N* = 117)	Hispanic (*N* = 377)	Other or unknown races (*N* = 157)^4^
All-cause mortality	*N* = 24	*N* = 11	*N* = 31	*N* = 8
Unadjusted model	Reference	1.64 (0.78, 3.45)	1.42 (0.81, 2.46)	0.85 (0.37, 1.93)
Adjusted model 1^1^	Reference	1.58 (0.74, 3.35)	1.75 (1.00, 3.09)	0.90 (0.39, 2.06)
Adjusted model 2^2^	Reference	1.13 (0.50, 2.52)	1.41 (0.77, 2.61)	0.71 (0.30, 1.70)
Adjusted model 3^3^	Reference	1.15 (0.52, 2.57)	1.41 (0.76, 2.61)	0.69 (0.29, 1.67)
Mechanical ventilation	*N* = 13	*N* = 5	*N* = 24	*N* = 3
Unadjusted model	Reference	1.34 (0.47, 3.84)	2.04 (1.02, 4.07)	0.59 (0.16, 2.08)
Adjusted model 1^1^	Reference	1.31 (0.45, 3.77)	2.55 (1.26, 5.18)	0.63 (0.18, 2.25)
Adjusted model 2^2^	Reference	1.22 (0.42, 3.56)	1.91 (0.93, 3.93)	0.55 (0.15, 1.97)
Adjusted model 3^3^	Reference	1.15 (0.38, 3.44)	2.02 (0.95, 4.30)	0.53 (0.15, 1.94)

^1^Adjusted model 1 was adjusted for age and sex

^2^Adjusted model 2 was adjusted for statistically selected variables (BMI, CCI score, cancer type, metastasis or secondary malignant neoplasm, recent cancer diagnoses in UC CORDS, and vaccination status for mortality; CCI score and vaccination status for mechanical ventilation) plus age and sex

^3^Adjusted model 3 was adjusted for age, sex, BMI, CCI score, cancer type, recent cancer diagnoses in UC CORDS, metastasis or secondary malignant neoplasm, COVID-19 vaccination status, and dominant SARS-COV-2 variant at COVID-19 diagnosis

^4^Black was combined with other or unknown races due to the small sample size

**Table 3:** Odds ratios and 95% CIs of specific races relative to the White by the outcomes of hospitalization or ER visits in GI cancer patients with SARS-CoV-2 infection in 30 days

Outcome	White (*N* = 403)	Asian or Pacific Islander (*N* = 117)	Black (*N* = 51)	Hispanic (*N* = 377)	Other or unknown races (*N* = 106)
COVID-19-related hospitalization or ER visits	*N* = 50	*N* = 23	*N* = 13	*N* = 96	*N* = 21
Unadjusted model	Reference	1.73 (1.00, 2.98)	2.42 (1.20, 4.84)	2.41 (1.66, 3.51)	1.74 (1.00, 3.06)
Adjusted model 1^1^	Reference	1.73 (1.00, 3.00)	2.52 (1.24, 5.09)	2.93 (1.98, 4.32)	1.91 (1.08, 3.38)
Adjusted model 2^2^	Reference	1.68 (0.95, 2.95)	2.21 (1.07, 4.57)	2.36 (1.58, 3.53)	1.83 (1.02, 3.29)
Adjusted model 3^3^	Reference	1.64 (0.92, 2.90)	2.26 (1.08, 4.70)	2.24 (1.48, 3.39)	1.80 (1.00, 3.26)

^1^Adjusted model 1 was adjusted for age and sex

^2^Adjusted model 2 was adjusted for statistically selected variables (CCI score and vaccination status) plus age and sex

^3^Adjusted model 3 was adjusted for age, sex, BMI, CCI score, cancer type, recent cancer diagnoses in UC CORDS, metastasis or secondary malignant neoplasm, COVID-19 vaccination status, and dominant SARS-COV-2 variant at COVID-19 diagnosis

In the stratified analysis, Hispanic patients had a 1.91-fold (OR = 1.91, 95% CI = 1.15–3.19) and 3.15-fold (OR = 3.15, 95% CI = 1.51–6.55) risk of COVID-19-related hospitalization or ER visits before and after the Omicron becoming the dominant SARS-COV-2 variant, in comparison to the White patients. Moreover, the two ORs for patients of other or unknown races were 1.68 (95% CI = 0.89–3.19) and 2.61 (95% CI = 1.11–6.16), respectively. The results of the stratified analysis are shown in Supplementary Table 2. We also analyzed the proportion of vaccination in these two periods (Supplementary Table 3). Results showed that only 8.3% of included GI cancer patients were fully vaccinated before Omicron became the dominant variant, while the proportion increased to 40.6% later.

### Risk Factors Associated with Clinical Prognosis

The multivariable logistic models illustrated the association between several demographic, health-related, cancer-associated factors and clinical prognosis of interest in GI cancer patients (Table 4). Age was a demographic factor significantly positively associated with all-cause mortality (OR = 1.04, 95% CI = 1.01–1.06), mechanical ventilation (OR = 1.03, 95% CI = 1.01–1.06), andCOVID-19-related hospitalization or ER visits (OR = 1.02, 95% CI = 1.01–1.03) within 30 days of SARS-COV-2 infection. CCI was another factor positively associated with risks of all adverse clinical outcomes. Compared with patients with a CCI score of 0–2, patients with a CCI score ≥ 3 had higher odds of death (OR = 3.11, 95% CI = 1.59–6.09), mechanical ventilation (OR = 3.89, 95% CI = 1.57–9.60) and COVID-19-related hospitalization or ER visits (OR = 2.52, 95% CI = 1.71–3.71). In addition, we found that fully vaccinated patients had more than 70% decrease in the odds of death (OR = 0.28, 95% CI = 0.10–0.77), mechanical ventilation (OR = 0.27, 95% CI = 0.08–0.94), or COVID-19-related hospitalization or ER visits (OR = 0.27, 95% CI = 0.15–0.49). As presented in Supplementary Table 3, the rate of full vaccination in all included patients increased substantially from 8.3 to 40.6% before and after the predominance of Omicron in December 2021. However, the increase was more minor among Hispanic patients (from 5.5 to 33.9%) and patients of other or unknown races (from 8.3 to 34.8%). Regarding cancer types, patients with hepatobiliary or pancreatic cancer had a significantly higher 30-day all-cause mortality than patients with GI tract cancer (OR = 1.84, 95% CI = 1.08–3.13). Metastasis or secondary malignant neoplasm was another cancer-related risk factor for mortality (OR = 2.79, 95% CI = 1.65–4.71). Furthermore, compared with patients with recent GI cancer diagnoses in UC CORDS, patients with GI cancer diagnoses recorded one more year before the infection had a decreased odds of death (OR = 0.57, 95% CI = 0.34–0.95) within 30 days of SARS-COV-2 infection. No other significant association was found between other variables and COVID-19 clinical outcomes.

**Table 4:** Adjusted odds ratios and 95% CIs of risk factors and clinical outcomes in GI patients within 30 days of SARS-CoV-2 infection

Variable	Death (*N* = 74)	Mechanical ventilation (*N* = 45)	COVID-19-related hospitalization or ER visits (*N* = 203)
Age, years	1.04 (1.01, 1.06)	1.03 (1.01, 1.06)	1.02 (1.01, 1.03)
BMI, kg/m^2^	0.95 (0.91, 1.00)	1.01 (0.96, 1.07)	1.00 (0.97, 1.03)
Sex			
Female	Reference	Reference	Reference
Male	1.21 (0.72, 2.04)	1.44 (0.75, 2.79)	1.34 (0.96, 1.88)
CCI score			
0–2	Reference	Reference	Reference
≥ 3	3.11 (1.59, 6.09)	3.89 (1.57, 9.60)	2.52 (1.71, 3.71)
Cancer type			
GI tract cancer	Reference	Reference	Reference
Hepatobiliary or pancreatic cancer	1.84 (1.08, 3.13)	1.32 (0.69, 2.52)	1.19 (0.85, 1.68)
Multiple GI cancers	1.87 (0.62, 5.70)	1.22 (0.25, 5.97)	1.07 (0.45, 2.57)
Other or ill-defined GI cancer	NA	NA	1.17 (0.21, 6.61)
Recent cancer diagnoses in UC CORDS			
Yes	Reference	Reference	Reference
No	0.57 (0.34, 0.95)	0.56 (0.30, 1.04)	0.82 (0.59, 1.14)
Metastasis or secondary malignant neoplasm			
No	Reference	Reference	Reference
Yes	2.79 (1.65, 4.71)	1.88 (0.98, 3.61)	0.90 (0.63, 1.28)
Dominant SARS-CoV-2 variant at COVID-19 diagnosis			
Original types before Delta	Reference	Reference	Reference
Delta	0.56 (0.20, 1.55)	0.65 (0.18, 2.31)	0.95 (0.54, 1.68)
Omicron	0.87 (0.49, 1.55)	1.09 (0.54, 2.20)	0.87 (0.60, 1.27)
Vaccination status			
Unvaccinated	Reference	Reference	Reference
Partially vaccinated	0.48 (0.11, 2.16)	NA	0.89 (0.40, 1.98)
Fully vaccinated	0.28 (0.10, 0.77)	0.27 (0.08, 0.94)	0.27 (0.15, 0.49)

All ORs were adjusted for race, age, sex, BMI, CCI score, cancer type, recent cancer diagnoses in UC CORDS, metastasis or secondary malignant neoplasm, COVID-19 vaccination status, and dominant SARS-COV-2 variant at COVID-19 diagnosis

*NA* not applicable

## Discussion

Racial disparities exist in the incidence and prevalence of GI cancer, and the COVID-19 pandemic might impact this situation. Due to the uniquely diverse racial-ethnic makeup in the population of California and the real-world medical information across the UC Health system, we conducted this retrospective study to fill the knowledge gap regarding racial disparities in the prognosis of GI cancer patients with COVID-19.

Racial disparities in hospitalizations or ER visits in GI cancer patients with COVID-19 were detected in this study. Compared to the non-Hispanic White patients, the Black patients, the Hispanic patients, and the patients of other or unknown races had an increased risk of COVID-19-related hospitalization or ER visits but no significant difference in all-cause mortality or ventilation. The disparities persisted across three analytical models, suggesting that more undetected biological or socioeconomic factors may contribute to our findings. Growing evidence has shown that African Americans demonstrate unfavorable tumor biology in colorectal cancer compared to White patients, such as a decreased fraction of macrophages and CD8 + T cells, which may cause worse cancer survival [21]. In addition, the frequencies of the fatty acid desaturase (FADS) gene haplotype are higher among African Americans and Hispanics, leading to a more efficient biosynthesis of long-chain polyunsaturated fatty acids from the omega-6 fatty acids in the diet, elevated level of cytokines, and higher vulnerabilities of these populations when confronted with SARS-COV-2 infection [22]. COVID-19 also highlighted the interaction of race and social determinants, such as access to medical care, living conditions, education level, and lifestyle choices. For instance, racial minorities make up 60% of warehouse and delivery workers and 74% of cleaning service workers, resulting in unequal challenges in social distancing, susceptibility, and disease outcomes [23]. A multicenter prospective study showed that 40.6% of cancer patients experienced fewer medical visits during the pandemic. Compared to White patients, Hispanic patients were more likely to experience visit reduction (OR = 1.34, 95% CI = 1.02–1.77) and then treatment delays (OR = 1.53, 95% CI = 1.03–2.26) [24]. Hispanic and Black patients were also less likely to use telehealth during this period [24]. Data from the Behavioral Risk Factor Surveillance System survey showed that colonoscopy screening percentages decreased by 4.1%, 2.3%, and 3.8% among AI/AN, Hispanic, and multiracial participants in 2020, respectively, compared with those in 2014–2019, while the percentage of the White participants dropped by 1.2% [25]. This may indicate higher reduced participation in disease prevention programs among racial minorities and may partially help to interpret our study results. To mitigate racial disparities, healthcare policies, education, employment, housing, and more that currently contribute to poor health in racial minorities should be adjusted and improved [26, 27]. Moreover, it is also essential to have racial and ethnic minorities be a part of the public health research design and collect socio-economic information or biological specimen alongside racial data in studies identifying remedies for racial disparities in COVID-19 [28, 29].

Similar racial disparities were reported in studies regarding other types of cancer or cancer in general. A COVID-19 and Cancer Consortium (CCC19) registry-based study showed that Black cancer patients suffered from significantly more severe cases of COVID-19 than White patients, measured by a 5-level ordinal scale including hospitalization, mechanical ventilation, and all-cause mortality [6]. Kathuria-Prakash et al. reported that Hispanic/Latinx ethnicity had a higher hospitalization rate among breast cancer patients with COVID-19 [7], and our results on GI cancer reflect a similar pattern. Also, based on the UC CORDS dataset, Kwon et al. identified Hispanic ethnicity as a risk factor for hospitalization but no racial/ethnical disparities in mechanical ventilation and death within 30 days following COVID-19 among cancer patients. Their results were consistent with our study on GI cancer specifically. However, our results differed slightly from Kwon’s study because we only included GI cancer patients, while they studied cancer patients in general. In addition, our study covered multiple COVID-19 waves and adjusted for vaccination status, which may explain the discrepancies in the results compared to other studies [30].

Even though the factors mentioned above might have contributed to the racial disparities, the odds of COVID-19 adverse outcomes in certain racial groups decreased after controlling for potential risk factors, which might directly or indirectly impact the differences in prognosis among all racial groups. Previously reported risk factors for patients with COVID-19, such as age and comorbidities [31, 32], were consistent in our study. However, we did not find an association between gender and COVID-19 clinical outcomes, which might be due to the small sample size [33, 34]. Regarding cancer elements, like earlier studies on COVID-19 and cancer in general, we also found a positive association between recent cancer diagnoses, metastatic cancer, and poor prognosis [5, 35]. Specifically among GI cancers, hepatobiliary or pancreatic cancer had higher odds of death within 30 days of SARS-COV-2 infection. In addition to the low 5-year survival rate for patients with hepatobiliary or pancreatic cancer, COVID-19 may aggravate the preexisting chronic liver disease and complicate cancer management through various mechanisms, such as direct viral entry into cells, hypoxia, immune-mediated hepatitis, and drug-associated hepatotoxicity [36]. Additionally, lower BMIs were associated with a worse prognosis which is consistent with the tendency of cachexia in the later stage of cancer progression.

Furthermore, vaccination status is a crucial factor associated with the prognosis of GI cancer patients among different race/ethnicity groups. A study has revealed significantly improved immunogenicity in fully vaccinated patients with solid cancer using the data from CCC19 [37]. Our study validated this data by showing that being fully vaccinated was significantly associated with around 70% reduced risk of outcomes among patients with GI cancer compared with unvaccinated patients. In contrast to the study by Schmidt et al. that reported no statistical difference in 30-day mortality between the fully vaccinated and the unvaccinated cancer patients [38], our study provides more robust results because we only included GI cancer patients and the full vaccination rate was 21.8%. In contrast, they had 1787 patients with various cancer types, and only 3% (54) were fully vaccinated. In addition, Poghosyan et al. found a higher COVID-19 vaccination rate in non-Hispanic White Medicare beneficiaries with cancer histories compared with that in the Black and Hispanic counterparts, which is consistent with our results [39]. Moreover, the percentage of fully vaccinated (66.7%) people in the US population before May 8, 2022, was much higher than that (21.8%) in the GI cancer patients included in our study [40]. This indicated vaccine hesitancy, defined as a “delay in acceptance or refusal of vaccination despite the availability of vaccination services” among cancer patients, especially those belonging to racial minorities [41]. According to a survey, cancer patients are mainly concerned about the side effects of vaccines, especially thrombosis, while unvaccinated patients would like to receive more straightforward medical advice [42]. Therefore, targeted communication emphasizing vaccine safety in the context of cancer may encourage vaccination uptake in this vulnerable population and further ameliorate the racial disparities as described [42]. Intriguingly, we also found that the disparities in hospitalization or ER visits became larger in racial minorities after Omicron became the dominant variant. The relatively low participation in the vaccination program in racial minorities may have been a factor.

### Limitations

This study has a few limitations, including the small sample size and its nature as a retrospective EHR-based study. As described above, the small sample size in certain racial groups or specific GI cancer types limits the ability to explore the racial disparities in 30-day all-cause mortality and the mechanical ventilation rate. The second limitation is the potentially incomplete capture of EHRs, which only cover the medical information of patients who seek healthcare in the UC Health system. Primary admission diagnoses and causes of mortality are also not precise and complete in the EHR. However, we included multiple outcomes to overcome the bias caused by the general frequency of hospital visits and make the results more robust. Furthermore, risk factors information is not available for all GI cancer patients (e.g., *H. pylori* information is available for most gastric cancer patients but not for the rest of the patients, and it is also hard to assess the impact of socioeconomic status and lifestyles due to the dearth of related data in EHRs. Despite these limitations, this multi-center real-world study, which covers all waves of COVID-19 so far and includes vaccination status as a covariate, still allows us to identify racial disparities and risks in clinical outcomes among GI cancer patients with COVID-19.

### Conclusions

Our study found that Black and Hispanic GI cancer patients had a higher risk of COVID-19-related hospitalization or ER visits within 30 days of SARS-COV-2 infection than non-Hispanic White patients. We observed no significant racial disparities in 30-day all-cause mortality and mechanical ventilation rate. Our study highlights the importance of vaccination in reducing the risk of adverse COVID-19 outcomes among GI cancer patients. Other factors, such as age, cancer type, recent cancer diagnoses in UC CORDS, metastatic cancer or secondary malignant neoplasm, and comorbidities, were also associated with the prognosis of GI cancer and COVID-19 and might have contributed to the racial disparities. To our knowledge, this study is the first to report racial disparities in clinical outcomes of GI cancer patients with COVID-19. More extensive studies are warranted to investigate further the underlying factors associated with such disparities.

### Supplementary Information

Below is the link to the electronic supplementary material.

Supplementary file1 (DOCX 31 KB)
