# Supplementary material for: Racial Disparities in the Clinical Prognosis of Gastrointestinal Cancer Patients with COVID-19: a Retrospective Study in UC CORDS
Source: J Racial Ethn Health Disparities. 2023 Jan 13;11(1):216–25. doi: 10.1007/s40615-023-01512-w (PMC9838534; doi:10.1007/s40615-023-01512-w)
Supplement: Supplementary file 1 — Supplementary file1 (DOCX 31 KB) [file 40615_2023_1512_MOESM1_ESM.docx]

**Supplementary Information**

**Article title:** Racial disparities of clinical prognosis in gastrointestinal cancer patients with COVID-19: a retrospective study in UC CORDS

**Journal name:** Journal of Racial and Ethnic Health Disparities

**Author names:** Bingya Ma, Yunxia Lu

**Affiliation:** Department of Population Health and Disease Prevention, Program in Public Health, Susan and Henry Samueli College of Health Sciences, University of California, Irvine, CA, USA

**E-mail:** Yunxia.lu@uci.edu

**Supplementary Table 1 (Sensitivity Analysis)**

Adjusted odds ratios and 95% CIs of specific races relative to the White by the outcomes and missing value status in GI cancer patients with SARS-COV-2 infection in 30 days

| Outcome and racial group | All included patients (N=1,054) | Excluding patients with missing height/weight values (N=1,009) | Excluding patients with missing height/weight values or weight values measured more than three months before COVID-19 diagnosis (N=821) |
| --- | --- | --- | --- |
| **All-cause mortality** | | | |
| White | Reference | Reference | Reference |
| Asian or Pacific Islander | 1.15 (0.52, 2.57) | 1.20 (0.53, 2.69) | 1.14 (0.49, 2.65) |
| Hispanic | 1.41 (0.76, 2.61) | 1.52 (0.81, 2.86) | 1.42 (0.74, 2.73) |
| Other or unknown races^1^ | 0.69 (0.29, 1.67) | 0.64 (0.25, 1.61) | 0.61 (0.23, 1.64) |
| **Mechanical ventilation** | | | |
| White | Reference | Reference | Reference |
| Asian or Pacific Islander | 1.15 (0.38, 3.44) | 1.18 (0.39, 3.53) | 1.34 (0.44, 4.09) |
| Hispanic | 2.02 (0.95, 4.30) | 2.04 (0.96, 4.35) | 2.18 (1.00, 4.77) |
| Other or unknown races^1^ | 0.53 (0.15, 1.94) | 0.55 (0.15, 1.99) | 0.63 (0.17, 2.33) |
| **COVID-19-related hospitalizations or ER visits** | | | |
| White | Reference | Reference | Reference |
| Asian or Pacific Islander | 1.64 (0.92, 2.90) | 1.57 (0.88, 2.81) | 1.49 (0.80, 2.79) |
| Black | 2.26 (1.08, 4.70) | 2.23 (1.07, 4.65) | 2.69 (1.21, 5.98) |
| Hispanic | 2.24 (1.48, 3.39) | 2.26 (1.49, 3.42) | 2.23 (1.43, 3.46) |
| Other or unknown races^1^ | 1.80 (1.00, 3.26) | 1.76 (0.96, 3.21) | 1.81 (0.94, 3.51) |

1 Black was combined with other or unknown races due to the small sample size.

**Supplementary Table 2**

Adjusted odds ratios and 95% CIs of specific races relative to the White by the outcomes in GI cancer patients with SARS-COV-2 infection in 30 days before and after Omicron as the dominant variant in California (December 20, 2021)

| Race | All-cause mortality | | Mechanical ventilation | | COVID-19-related hospitalizations or ER visits | |
| --- | --- | --- | --- | --- | --- | --- |
|  | N | OR (95%CI)^2^ | N | OR (95%CI)^2^ | N | OR (95%CI)^2^ |
| **Before Omicron was the dominant variant** | | | | | | |
| White | 15 | Reference | 9 | Reference | 33 | Reference |
| Asian or Pacific Islander | 6 | 0.88 (0.30, 2.61) | 3 | 0.83 (0.20, 3.45) | 14 | 1.54 (0.73, 3.25) |
| Hispanic | 21 | 1.32 (0.61, 2.88) | 16 | 1.40 (0.56, 3.51) | 67 | 1.91 (1.15, 3.19) |
| Other or unknown races^1^ | 7 | 1.00 (0.36, 2.79) | 1 | 0.21 (0.03, 1.74) | 22 | 1.68 (0.89, 3.19) |
| **After Omicron was the dominant variant** | | | | | | |
| White | 9 | Reference | 4 | Reference | 17 | Reference |
| Asian or Pacific Islander | 5 | 1.53 (0.43, 5.51) | 2 | 1.20 (0.19, 7.45) | 9 | 1.63 (0.63, 4.20) |
| Hispanic | 10 | 1.40 (0.48, 4.06) | 8 | 3.83 (0.99, 14.84) | 29 | 3.15 (1.51, 6.55) |
| Other or unknown races^1^ | 1 | 0.20 (0.02, 1.81) | 2 | 1.57 (0.25, 9.80) | 12 | 2.61 (1.11, 6.16) |

1 Black was combined with other or unknown races due to the small sample size.

2 All ORs were adjusted for age, sex, BMI, CCI score, cancer type, recent cancer diagnoses in UC CORDS, metastasis or secondary malignant neoplasm, COVID-19 vaccination status, and dominant SARS-COV-2 variant at COVID-19 diagnosis.

**Supplementary Table 3**

Comparison of vaccination status in GI cancer patients with SARS-COV-2 infection before and after the predominance of Omicron in California (December 20, 2021)

| Vaccination status | Total | White | Asian or Pacific Islander | Black | Hispanic | Other or unknown races |
| --- | --- | --- | --- | --- | --- | --- |
| **Before the predominance of Omicron** | | | | | | |
| Unvaccinated | 546 (89.1%) | 176 (83.4%) | 51 (91.1%) | 27 (90.0%) | 238 (93.0%) | 54 (90.0%) |
| Partially vaccinated | 16 (2.6%) | 8 (3.8%) | 3 (5.4%) | 0 (0.0%) | 4 (1.6%) | 1 (1.7%) |
| Fully vaccinated | 51 (8.3%) | 27 (12.8%) | 2 (3.6%) | 3 (10.0%) | 14 (5.5%) | 5 (8.3%) |
| **After the predominance of Omicron** | | | | | | |
| Unvaccinated | 232 (52.6%) | 93 (48.4%) | 31 (50.8%) | 10 (47.6%) | 72 (59.5%) | 26 (56.5%) |
| Partially vaccinated | 30 (6.8%) | 14 (7.3%) | 2 (3.3%) | 2 (9.5%) | 8 (6.6%) | 4 (8.7%) |
| Fully vaccinated | 179 (40.6%) | 85 (44.3%) | 28 (45.9%) | 9 (42.9%) | 41 (33.9%) | 16 (34.8%) |
